# Hepatic adenoma regression after bariatric surgery: a case series and systematic review

**DOI:** 10.1007/s00464-025-12350-8

**Published:** 2025-11-10

**Authors:** Ian C. Garbarine, Amanda K. Walsh, Timothy M. Pawlik, Sabrena F. Noria, Jordan M. Cloyd

**Affiliations:** 1 Department of Surgery, The Ohio State University Wexner Medical Center, 410 W 10 Ave, N943 Doan Hall, Columbus, OH 43210 USA; 2 Division of Surgical Oncology, The Ohio State University Wexner Medical Center, 410 W 10th Ave, N907 Doan Hall, Columbus, OH 43210 USA; 3 Division of General and Gastrointestinal Surgery, Department of Surgery, 410 W 10th Ave N718 Doan Hall, Columbus, OH 43210 USA

**Keywords:** Hepatic adenoma, Bariatric surgery, Regression, HA, Roux-en-Y gastric bypass, Gastric sleeve

## Abstract

**Background:**

Hepatic adenomas (HA) are benign neoplasms of the liver that have small risks of hemorrhage and malignant transformation. While the association between obesity and the development of HAs is increasingly recognized, the impact of bariatric surgery on HA regression is poorly understood.

**Methods:**

All patients with a pre-operative diagnosis of HA who underwent primary bariatric surgery (sleeve gastrectomy or Roux-en-Y gastric bypass) at a single quaternary academic medical center from 2012 to 2024 were retrospectively queried and combined with all previously reported cases obtained via a systematic review of the literature. Patient clinical characteristics, including pre-operative and post-operative body mass index (BMI) and HA size, were extracted. Kendall’s Tau-b correlation and fractional probit regression were used to assess the relationship between weight loss and HA size change.

**Results:**

The institutional review identified three patients, and the systematic review yielded five studies totaling seven patients. In the combined cohort (mean age 34.8 years), the average pre-operative BMI was 44.48 kg/m^2^ and decreased to 32.19 kg/m^2^ post-operatively, representing 68.18% excess weight loss (%EWL). Mean HA size decreased from 4.49 cm to 1.55 cm (56.46% reduction, SD 43.68), with 40% of patients experiencing complete regression. %EWL was significantly associated with HA size reduction (Kendall’s Tau-b: 0.5528; *p* = 0.047). Marginal effects following fractional probit regression analysis showed that each additional percentage increase in %EWL was associated with 0.76% HA size reduction (95% CI: -1.18, -0.34; p < 0.001).

**Conclusions:**

In this combined case series and systematic review of the literature, bariatric surgery was associated with high rates of HA regression. Given the additional benefits of weight loss from bariatric surgery and the significant morbidity associated with liver resection, bariatric surgery could be considered as an initial management option for obese patients with HA.

**Supplementary Information:**

The online version contains supplementary material available at 10.1007/s00464-025-12350-8.

Hepatic adenomas (HA) are common benign neoplasms of the liver most often diagnosed in young women. Long-term use of estrogen-containing oral contraceptive pills (OCPs) has been associated with an approximately 30–40-fold increased risk of developing HAs [1, 2]. In these settings, withdrawal of estrogen-containing OCPs can lead to tumor stability or regression [3]. Additionally, patients who take exogenous steroids (e.g., for treatment of aplastic anemia, hereditary angioedema, or gender-affirming care) or who have increased endogenous sex hormone production (e.g., metabolic syndrome, polycystic ovarian syndrome) have a higher incidence of HAs, supporting the influence of hormone levels on HA development [4, 5]. Obesity, particularly in females, can lead to functional hyperandrogenism, as obese patients have increased estrogen levels compared with normal-weight counterparts due to increased rates of aromatization of androgens by adipocytes [6–8].

Although benign, HAs have the potential for hemorrhage as well as malignant transformation, the latter occurring in approximately 5% of patients [9]. HAs can be subdivided into at least eight subtypes based on molecular profiles, which aids in risk stratification and prognostication [9]. Current guidelines recommend the resection of HAs that are either symptomatic or > five cm in diameter, as larger lesions are associated with an increased risk of malignant transformation and hemorrhage [10]. Although hepatic resection for HA is generally recognized as safe, especially at experienced centers, it carries a risk of major post-operative complications nearing 10%—20% and impaired long-term quality of life [11–14].

The prevalence of obesity in the United States has increased significantly over the past three decades [15]. Specifically, the percentage of adults who qualify as obese increased from 16.7% in 1996 to 42.4% in 2018 [16]. Interestingly, while the use of estrogen-containing OCPs has decreased over the past three decades, rates of HAs have increased over this same period [17]. Several large retrospective case series have further established obesity as a risk factor for HAs, potentially mediated in part by increased circulating levels of interleukin-6 (IL-6) [17–19]. Given the association between obesity and HA, as well as the risks associated with hepatic resection, lifestyle management, including weight loss and cessation of OCPs, are often recommended initially for obese patients with HA. Bariatric surgery, including sleeve gastrectomy (SG) and Roux-en-Y gastric bypass (RYGB), is an attractive intervention for obese patients with symptomatic or large HAs, as significant and sustained weight loss using conservative efforts is uncommon [20, 21]. In fact, bariatric surgery is the most efficacious treatment for obesity because it provides the highest rates of sustained weight loss and resolution of comorbidities [15, 22, 23].

Currently, the evidence supporting bariatric surgery as a management option for HAs is sparse and consists of several small case reports and series [24–27]. Therefore, this study sought to leverage a single institution’s robust experience with bariatric surgery to evaluate the association between weight loss following bariatric surgery and HA regression at the institutional level. We also combined these findings with a systematic review and analysis of the current literature.

## Materials and methods

### Case series

This is a retrospective case series of adult patients from a single, quaternary academic medical center between March 2012 and January 2024. The study followed STROBE guidelines (Supplementary Table 1) and was approved by the Institutional Review Board [28]. Inclusion criteria included age > 16 years, patients who underwent either SG or RYGB, had evidence of HA prior to bariatric surgery, and had post-operative abdominal imaging at least six months after their index bariatric surgery. Patients who underwent hepatic resection, as well as patients without both pre- and post-operative imaging, were excluded.

Female patients who underwent bariatric surgery within the study’s timeframe were identified from the institution’s Metabolic and Bariatric Surgery Accreditation and Quality Improvement Program (MBSAQIP) database. Medical records, including radiology and pathology reports, were screened for ICD codes and keywords for liver lesions (Supplementary Table 2). These records were then independently reviewed by the authors for evidence of HAs on pre-operative imaging. Only patients who had evidence of HA on pre-operative as well as post-operative imaging were included.

### Systematic review

Given the lower-than-expected number of patients meeting inclusion criteria in our institutional series, a systematic review was also performed to identify publications reporting changes in HA size after bariatric surgery. PubMed, Embase, Cochrane Library, and Scopus databases were queried using the following search terms: *liver cell adenoma, liver adenoma, adenoma, hepatic adenoma, hepatocellular adenoma, benign hepatoma, telangiectatic focal nodular hyperplasia; bariatric surgery, gastrectomy, gastric bypass, gastric bypass surgery, gastric sleeve, sleeve gastrectomy, weight loss surgery*. Multiple combinations of search terms were used, and both free text and controlled vocabulary (MeSH and Emtree) were included. The initial search was completed on November 11, 2024.

All English language studies that examined patients who underwent primary bariatric surgery and had evidence of HA, including its size before and after surgery, were included. Studies investigating pharmaceutical and/or lifestyle interventions without surgical intervention were excluded, as were studies reporting patients who underwent hepatic resection only. Reviews, letters, commentaries, narratives, and study protocols were also excluded. Abstracts were allowed if there were no full-text duplicates. No restrictions were placed on the date of publication. Additional relevant studies were identified through manual searches of the literature.

Initial screening of eligible studies was performed through a review of titles and abstracts, followed by a secondary screening of the full texts. Each phase of screening was conducted by two independent reviewers (AKW, ICG), and disagreements were settled by consensus. The literature search and screening were completed using Covidence (Melbourne, Australia). The systematic review was performed according to PRISMA guidelines.

### Data extraction

Data extraction from the included studies was undertaken by two authors (AKW, ICG). Variables extracted included publication year, country of origin, and study type. Patient data extracted included gender, age, sex, pre- and post-operative BMI, pre-and post-operative hepatic adenoma size, follow-up interval, type of bariatric surgery performed, diagnostic and follow-up imaging modality, and pathology reports as applicable. Hepatic adenoma sizes were determined by greatest dimensions seen on cross-sectional imaging or ultrasound unless intraoperative measurements were noted.

### Quality appraisal

Each study was assessed for quality and presence of bias using the Joanna Briggs Institute (JBI) Critical Appraisal checklists by two independent reviewers (AKW, ICG), and disagreements were settled by consensus [29]. The JBI checklists for case reports and case series contain a series of “yes”, “no”, “unclear”, or “not applicable” questions to evaluate study design. Cutoffs at 70% and 50% of questions answered “yes” were used to determine high- and medium-quality studies, respectively. No studies were omitted based on quality assessment to ensure the entire body of literature was represented in this review.

### Statistical analysis

Percentage of excess weight loss (% EWL) was calculated for each patient based on the following formula: $\% EWL=\frac{Pre-operative BMI-Post-operative BMI}{Pre-operative BMI-Ideal BMI}\times 100$. For these calculations, ideal BMI was set at 25 kg/m^2^. Scatter plots with Kendall-Theil-Sen estimators, given the bounded and non-parametric nature of this data set, were used to visualize the data. For non-parametric data, Kendall’s Tau-b was calculated as appropriate. Fractional regression with probit link was also conducted. Regression coefficients and effect sizes in the probit model are not easily interpreted. Therefore marginal effects representing the change in the size of hepatic adenomas using the delta method to calculate 95% confidence intervals were performed. All *p* values used in the analyses were two-tailed, and *p* values < 0.05 were considered significant. Data were analyzed by ICG from November 2024 to January 2025 using Stata 18 (Stata Corporation, College Station, TX).

## Results

### Institutional cohort

**Fig. 1:**
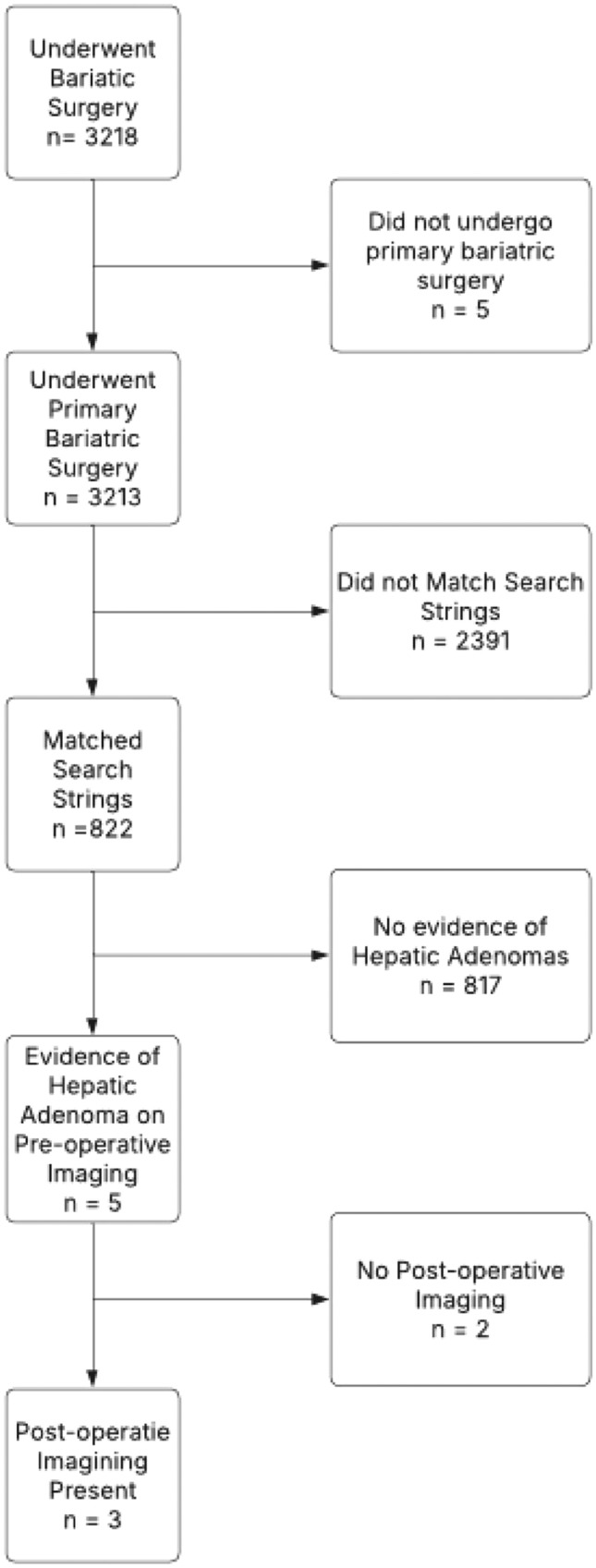
Flow chart of patients included in institutional case series

Among the 3,218 initial records retrieved, 827 unique patient charts were reviewed, ultimately resulting in five patients who underwent bariatric surgery and had evidence of HA on pre-operative imaging. Two records did not include post-operative cross-sectional imaging and were therefore excluded (Fig. 1). Patient characteristics of the institutional cohort are shown in Table 1.

**Table 1:** Institutional cohort characteristics

Age	Sex	Bariatric Surgery Type	Pre-op BMI (kg/m^2^)	Pre-op HA (cm)	Post-op BMI (kg/m^2^)	Post-op HA (cm)	%EWL	Imaging Interval (months)
37	F	RYGB	46.92	1.00	29.87	0	77.8%	36
42	F	RYGB	38.03	1.80	23.73	0	109.0%	48
35	F	SG	40.76	1.00	36.20	1.00	28.9%	7.5

Patient A, a 37-year-old female with pre-operative BMI of 46.92 kg/m^2^ underwent RYGB. She was noted to have three hepatic adenomas, each approximately one cm on MRI obtained due to right flank pain approximately one year prior to her bariatric surgery. The operation was uneventful; no obvious concerning changes to the hepatic surface were noted intraoperatively. Approximately 1.5 years after surgery, her BMI had decreased to 29.87 kg/m^2^. At that time, a computed tomography (CT) abdomen and pelvis (A/P) scan with contrast was obtained due to persistent nausea and emesis and showed no evidence of hepatic lesions.

Patient B, a 42-year-old female with a pre-operative BMI of 38.03 kg/m^2^, underwent RYGB. MRI obtained three years prior to her bariatric surgery showed a single HA measuring 1.8 cm × 1.4 cm in segment II, which was stable on CT immediately prior to her RYGB. The patient’s BMI decreased to 23.73 kg/m^2^ at 16 months post-operatively. Cross-sectional imaging obtained at the 16-month interval showed resolution of her HA.

Patient C, a 35-year-old female with a pre-operative BMI of 40.76 kg/m^2^, underwent sleeve gastrectomy. On pre-operative CT, a small 1.3 cm lesion was identified in segment IVb. MRI 18 months after SG showed a 1.1 cm HA in the same area as well as a small hepatic hemangioma in segment VII. The patient’s BMI at the time of her most recent MRI was 36.2 kg/m^2^.

### Systematic review cohort

**Fig. 2:**
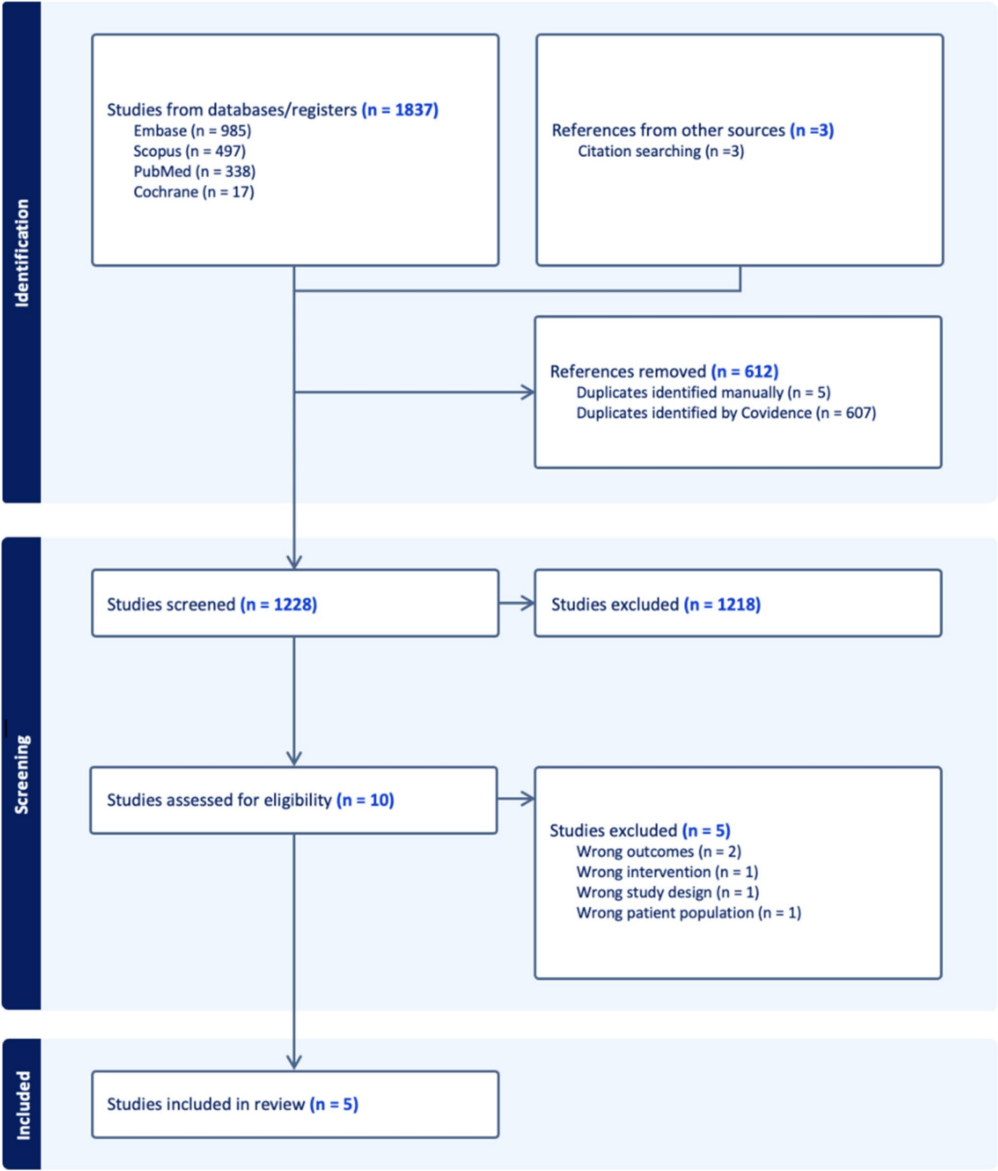
Study selection and flow PRISMA diagram for systematic review

Among the 1,840 publications retrieved in the initial query, including three from searching the reference list of relevant studies, 612 were identified as duplicates. Titles and abstracts were screened for the remaining 1,228 studies. Ultimately, after a full-text review of 10 articles, results were extracted from five studies that met inclusion criteria (Fig. 2). The literature search identified four case reports and one case series. All studies were from different countries, including the United Kingdom, Brazil, Qatar, the Netherlands, and France. Of the five studies included in the review, all had reporting rates of > 75%, with 4/5 studies having 100% complete reporting rates per JBI guidelines (Supplementary Table 3). From these studies, seven patients were identified, all of whom were female (Table 2). Four patients underwent RYGB, two underwent SG, and one underwent gastric banding.

**Table 2:**
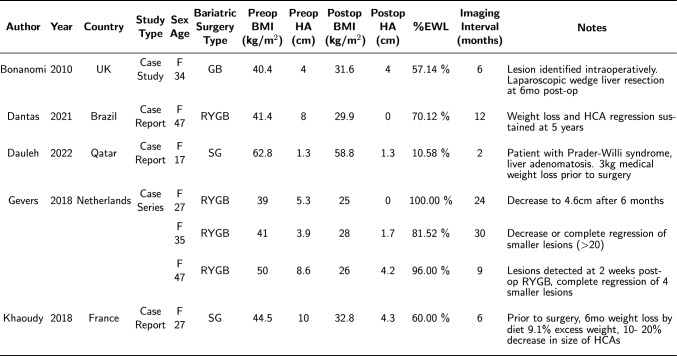
Systematic review patient characteristics

*GB* gastric banding, *SG* sleeve gastrectomy, *RYGB* Roux-en-Y gastric bypass

Gevers et al. (2018) described a case series of three obese patients with known HA who underwent RYGB. In all cases, patients lost significant excess body weight (31–48%) post-operatively and showed decreased size, or complete resolution, of their HA based on follow-up cross-sectional imaging [25].

Khaoudy et al. (2018) described the case of a 27-year-old female with a BMI of 46.5 kg/m^2^ and biopsy-proven HA (inflammatory subtype) noted to be 11 cm × 8.1 cm. This lesion was located near the middle hepatic vein, and resection would have required a right or central hepatectomy. This patient initially trialed medical weight loss with a decrease of 9.1% of excess weight, corresponding to a slight reduction in HA size to 10 cm × 7.7 cm. Given the persistently large size of the hepatic adenoma and elevated body weight, the patient subsequently underwent sleeve gastrectomy to provide additional weight loss and tumor control. Post-operatively, the patient lost 63.6% of excess weight with a corresponding decrease in adenoma size to 4.3 cm × 3.7 cm on follow-up imaging [26].

Bonanomi et al. (2008) described the case of a 34-year-old female with a BMI of 40.4 kg/m^2^ who underwent gastric banding [30]. Intraoperatively, the patient was noted to have a four cm nodule in segment III. An intraoperative biopsy of this lesion was performed, but the results were inconclusive, as histologic features could not differentiate between HA and focal nodular hyperplasia. The patient’s initial post-operative MRI suggested that the lesion identified intraoperatively was likely an HA. The patient discontinued oral contraceptives and proceeded to lose 24 kg in the subsequent six months for a 57.4% decrease in excess weight to 31.6 kg/m^2^. Follow-up MRI six months after gastric banding did not show any decrease in the size of the HA. The patient subsequently underwent laparoscopic hepatic resection, with final pathology of the surgical specimen consistent with HA.

Dantas et al. (2021) described the case of a 47-year-old female with a pre-bariatric surgery BMI of 41.4 kg/m^2^ who was noted to have a large, eight cm hepatic adenoma between segments VII and VIII found on routine abdominal ultrasound [31]. The patient subsequently underwent an uneventful RYGB. The patient initially maintained a 70.3% reduction in excess weight at one year with a concomitant resolution of the HA. While the patient regained weight between years one and five post-operatively, with a decrease to 66.5% reduction in excess weight at year five post-operatively, MRI obtained at that time did not have evidence of HA.

Dauleh et al. (2022) described the case of a 17-year-old female with Prader-Willi syndrome, a BMI of 64.4 kg/m^2^ who presented with liver adenomatosis (≥ 10 HAs), the largest measuring 1.3 cm in diameter [32]. Despite numerous attempts at non-surgical weight loss including diet, exercise, and a trial of glucagon-like peptide 1 receptor (GLP-1) agonist, the patient experienced persistent weight gain. She subsequently underwent a sleeve gastrectomy with a resulting 7.8 kg weight loss two months post-operatively without change in the sizes of her HAs.

### Combined cohort

The combined institutional and systematic review cohort consisted of 10 female patients with an average age of 34.8 years, ranging from 17 to 47 years old at the time of bariatric surgery. The average (SD) pre-operative BMI was 44.48 (7.42) kg/m^2^, and the average post-operative BMI was 32.19 (10.08) kg/m^2^, corresponding to an average (SD) reduction in BMI after surgery of 28.41 (12.60) % and mean (SD) %EWL of 68.18 (9.49) %. The mean size of the largest HA was 4.49 cm pre-operatively and 1.55 cm post-operatively (mean (SD) change in size -56.46% (43.68)).

**Fig. 3:**
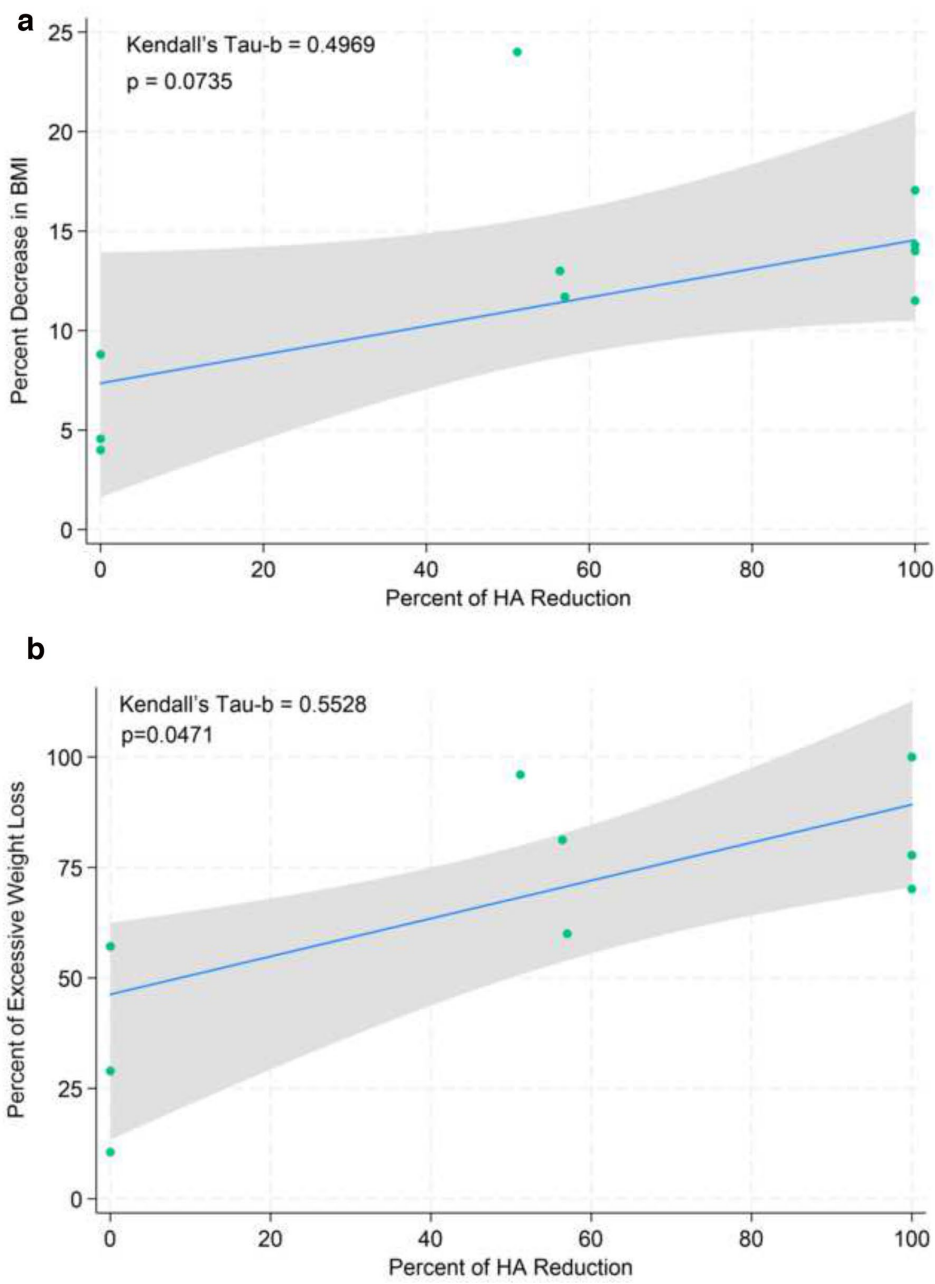
**a**: Scatter plot of percent reduction in HA size vs percent decrease in BMI after bariatric surgery. The blue line represents the Kendall-Theil-Sen estimator. The gray shaded area represents the 95% confidence interval. **b**: Scatter plot of percent reduction in HA size vs percent of excessive weight loss after bariatric surgery. The blue line represents the Kendall-Theil-Sen estimator. The gray shaded area represents the 95% confidence interval

To visualize the association between the change in BMI and the percent change in HA size after bariatric surgery, a scatter plot was created, and a Kendall-Thiel-Sen estimator was fitted to the data (Fig. 3a). A similar plot was created to visualize %EWL vs percent change in HA size (Fig. 3b). Weight loss, as defined as percent decrease in BMI, was not statistically significantly associated with percent reduction in HA size (Kendall’s Tau-b: 0.4969; p = 0.0735), although it trended toward significance. However, changes in %EWL were statistically significantly associated with percent reduction in HA size (Kendall’s Tau-b: 0.5528; p = 0.0471). Fractional probit regression analysis was utilized to analyze variables predictive of changes in HA size post-operatively. In this model, %EWL is associated with decreased HA size (p = 0.004; 95% CI -7.46, -1.45). Average marginal effects were then calculated, suggesting that each additional percent increase in %EWL is associated with 0.76% HA size reduction (95% CI: -1.18, -0.34; p < 0.001) when holding age constant at the mean for the population (Table 3).

**Table 3:** Fractional regression model of change in hepatic adenoma size

	Regression Coefficient	Robust Standard Error	95% CI	P value
%EWL	-4.46	1.53	-7.46, -1.45	0.004^∗^
Age	0.007	0.052	-0.096, 0.109	0.896
**Marginal Effects**				
	Marginal Effect	95% CI	P value	
%EWL	-0.763	-1.18, -0.34	< 0.001^∗^	
Age	0.06	-0.84, 0.96	0.898	

^*^Denotes statistical significance at a p < 0.05 significance level

## Discussion

Despite decreased use of oral estrogen-containing contraceptives, the prevalence of HAs has not declined correspondingly, underscoring the role of obesity in HA formation [4, 33]. Given the established association between HA formation and obesity, interventions aimed at weight loss could represent a rational initial therapeutic approach for many patients with HAs. Nevertheless, this systematic review of the literature identified a relative scarcity of published series on the role of weight-loss surgery among patients with HAs. Combined with data from our own institutional series, we noted that the percentage of excess weight loss (%EWL) following bariatric surgery was significantly associated with change in HA size. Notably, 40% of patients experienced complete regression of their HA. Given the additional benefits of weight loss from bariatric surgery and the significant morbidity associated with liver resection, bariatric surgery could be a promising strategy for obese patients with HA.

While benign, HAs maintain the potential for both malignant transformation and hemorrhage. For this reason, surgical resection has traditionally been offered to patients who meet specific clinical criteria, such as the presence of symptomatic HA or lesions that are greater than five cm in diameter [10]. Given the morbidity associated with hepatic surgery even in generally young, healthy women, there has been considerable interest recently in nonoperative management. For example, current guidelines now typically recommend cessation of OCPs as the preferred initial management even for HAs that meet resection criteria [34]. However, for obesity-related HA, evidence supporting the role of weight loss as a management strategy is less robust [27, 35]. In a large institutional series of non-surgical management of HA (including OCP cessation, medical weight loss through calorie reduction as well as exercise, and arterial embolization) in patients with a BMI ≥ 35 kg/m^2^, only two patients experienced regression of their HAs with diet and exercise alone. Both patients had significant weight loss (31% reduction in BMI) [27]. Along with the results of our study, this finding suggests that dramatic changes in %EWL are necessary to induce regression of HA. Indeed, the few patients in our study who did not achieve a reduction in their HA size, experienced minimal weight loss during the follow-up period.

The exact mechanism of HA regression after bariatric surgery is unknown. However, the reduction of circulating androgens and improved metabolic profile after bariatric surgery likely contribute. Bariatric surgery is associated with decreased hepatic steatosis, thought to be secondary to reduced hepatic inflammation, improved insulin sensitivity, and improved gut microbiota [36–40]. The reduction of persistent inflammation of hepatocytes, improved hepatic metabolism, and decrease in systemic inflammation through an improved hormonal milieu likely promotes regression of HAs. Over the past several years, there has been an inundation of new, effective weight-loss medications on the market. These glucagon-like peptide-1 (GLP-1) receptor agonists function by stimulating endogenous insulin release, inhibiting gastric emptying, and inhibiting endogenous glucagon release [41]. Given the relatively recent approval of these drugs for weight loss, no studies to date have investigated their impact on HAs. However, existing evidence from animal models and retrospective studies in patients with diabetes demonstrates a decrease in transaminitis and hepatic steatosis with use of GLP-1 receptor agonists [42, 43]. This suggests that GLP-1 receptor agonists may be beneficial for treating HAs through multiple mechanisms. By promoting weight loss, these agents may reduce levels of circulating androgens, which are implicated in HA pathogenesis. Additionally, obesity can lead to a constitutively pro-inflammatory state characterized by elevated IL-6 production. High levels of IL-6, in the setting of chronic inflammation, can contribute to HA formation, specifically the inflammatory subtype of HA [4, 44]. The reduction of hepatic steatosis seen with GLP-1 receptor agonist use, potentially through a reduction in hepatocyte sensitivity to circulating pro-inflammatory cytokines or reduced levels of these cytokines, may support HA regression, although more research is needed to elucidate these pathways fully.

Despite the large volume of young obese women undergoing primary bariatric surgery at our institution, a large quaternary medical center, the prevalence of HAs was relatively low during the study period (i.e. only 0.61% of female patients). Within the institutional cohort, only five patients were noted to have HA prior to bariatric surgery, and only three of those patients had appropriate post-operative imaging within the follow-up period. As patients undergoing bariatric surgery are in themselves a selected group, further research is needed to estimate the incidence of HA among obese persons overall.

While our study suggests a benefit of bariatric surgery and weight loss in general for HA regression, the routine use of bariatric surgery for the management of obesity-associated HAs may be challenging. Previous research has highlighted the barriers to bariatric surgery, including lower socioeconomic status, non-English language as the primary language, rural residence, uninsured or underinsurance status, and older age [45–49]. Strategies will be needed to overcome these barriers for bariatric surgery to be a feasible option for managing HAs.

A strength of our study, despite its small sample size, is the systematic review and inclusion of all published reports on this topic. Along with our own case series, we report the largest combined cohort of patients with HA undergoing bariatric surgery. Still, several limitations should be acknowledged. Due to the study design, follow-up time, imaging modality, type of bariatric surgery, indications for treatment, and more were not standardized across the cohort. In addition, given the nonrandomized nature of the study, there may be other confounding factors, and a causative relationship between bariatric surgery and HA regression cannot be definitely concluded. Finally, information on HA subtype was not available, and therefore the association between weight loss and HA regression in different subtypes requires further investigation.

In conclusion, using a combined institutional series and systematic review of existing literature, we report the largest series of patients with HA undergoing bariatric surgery. A strong association between %EWL after bariatric surgery and HA regression was observed, suggesting that bariatric surgery could be considered as an initial management option for patients with obesity-related HA. Future prospective trials investigating the role of intentional weight loss for hepatic adenoma regression are warranted.

## Supplementary Information

Below is the link to the electronic supplementary material.

Supplementary file1 (DOCX 35 KB)

Supplementary file2 (DOCX 28 KB)

Supplementary file3 (DOCX 21 KB)

## References

[1] Rooks JB, Ory HW, Ishak KG, Strauss LT, Greenspan JR, Hill AP, Tyler CW Jr (1979) Epidemiology of hepatocellular adenoma. The role of oral contraceptive use. JAMA 242:644–648 221698

[2] Edmondson HA, Henderson B, Benton B (1976) Liver-cell adenomas associated with use of oral contraceptives. N Engl J Med 294:470–472 173996 10.1056/NEJM197602262940904

[3] Haring MPD, Gouw ASH, de Haas RJ, Cuperus FJC, de Jong KP, de Meijer VE (2019) The effect of oral contraceptive pill cessation on hepatocellular adenoma diameter: a retrospective cohort study. Liver Int 39(5):905–913 30773766 10.1111/liv.14074 PMC6593966

[4] Tsilimigras DI, Rahnemai-Azar AA, Ntanasis-Stathopoulos I, Gavriatopoulou M, Moris D, Spartalis E, Cloyd JM, Weber SM, Pawlik TM (2019) Current approaches in the management of hepatic adenomas. J Gastrointest Surg 23:199–209 30109469 10.1007/s11605-018-3917-4

[5] Vijay A, Elaffandi A, Khalaf H (2015) Hepatocellular adenoma: an update. World J Hepatol 7:2603–2609 26557953 10.4254/wjh.v7.i25.2603 PMC4635146

[6] Pasquali R (2006) Obesity and androgens: facts and perspectives. Fertil Steril 85:1319–1340 16647374 10.1016/j.fertnstert.2005.10.054

[7] Ylli D, Sidhu S, Parikh T, Burman KD (2000) Endocrine changes in obesity. In: Feingold KR, Anawalt B, Blackman MR, Boyce A, Chrousos G, Corpas E, de Herder WW, Dhatariya K, Dungan K, Hofland J, Kalra S, Kaltsas G, Kapoor N, Koch C, Kopp P, Korbonits M, Kovacs CS, Kuohung W, Laferrère B, Levy M, McGee EA, McLachlan R, New M, Purnell J, Sahay R, Shah AS, Singer F, Sperling MA, Stratakis CA, Trence DL, Wilson DP (eds) Endotext. South Dartmouth, MA: MDText.com

[8] Bélanger C, Luu-The V, Dupont P, Tchernof A (2002) Adipose tissue intracrinology: potential importance of local androgen/estrogen metabolism in the regulation of adiposity. Horm Metab Res 34:737–745 12660892 10.1055/s-2002-38265

[9] Klompenhouwer AJ, de Man RA, Dioguardi Burgio M, Vilgrain V, Zucman-Rossi J, Ijzermans JNM (2020) New insights in the management of hepatocellular adenoma. Liver Int 40:1529–1537 32464711 10.1111/liv.14547 PMC7383747

[10] European Association for the Study of the Liver (2016) EASL clinical practice guidelines on the management of benign liver tumours. J Hepatol. 65:386–398 27085809 10.1016/j.jhep.2016.04.001

[11] Hoffmann K, Unsinn M, Hinz U, Weiss KH, Waldburger N, Longerich T, Radeleff B, Schirmacher P, Büchler MW, Schemmer P (2015) Outcome after a liver resection of benign lesions. HPB (Oxford) 17:994–1000 26456947 10.1111/hpb.12496 PMC4605338

[12] Newhook TE, LaPar DJ, Lindberg JM, Bauer TW, Adams RB, Zaydfudim VM (2016) Morbidity and mortality of hepatectomy for benign liver tumors. Am J Surg 211:102–108 26307421 10.1016/j.amjsurg.2015.06.010

[13] Laurent A, Dokmak S, Nault JC, Pruvot FR, Fabre JM, Letoublon C, Bachellier P, Capussotti L, Farges O, Mabrut JY, Le Treut YP, Ayav A, Suc B, Soubrane O, Mentha G, Popescu I, Montorsi M, Demartines N, Belghiti J, Torzilli G, Cherqui D, Hardwigsen J (2016) European experience of 573 liver resections for hepatocellular adenoma: a cross-sectional study by the AFC-HCA-2013 study group. HPB (Oxford) 18:748–755 27593592 10.1016/j.hpb.2016.06.011 PMC5011084

[14] Armstrong EA, Ejaz A, Sarna A, Conteh L, Tsung A, Pawlik TM, Cloyd JM (2020) A cross-sectional evaluation of quality of life among patients with hepatic adenomas. J Gastrointest Surg 24:2862–2864 32865734 10.1007/s11605-020-04780-7

[15] Sebastian R, Howell MH, Chang KH, Adrales G, Magnuson T, Schweitzer M, Nguyen H (2019) Robot-assisted versus laparoscopic Roux-en-Y gastric bypass and sleeve gastrectomy: a propensity score-matched comparative analysis using the 2015–2016 MBSAQIP database. Surg Endosc 33:1600–1612 30225604 10.1007/s00464-018-6422-7

[16] Hales CM, Carroll MD, Fryar CD, Ogden CL (2020) Prevalence of obesity and severe obesity among adults: United States, 2017–2018. NCHS Data Brief. 1–8 32487284

[17] Bioulac-Sage P, Taouji S, Possenti L, Balabaud C (2012) Hepatocellular adenoma subtypes: the impact of overweight and obesity. Liver Int 32:1217–1221 22429502 10.1111/j.1478-3231.2012.02786.x

[18] Bröker MEE, Gaspersz MP, Klompenhouwer AJ, Hansen BE, Terkivatan T, Taimr P, Dwarkasing R, Thomeer MGJ, de Man RA (2017) Inflammatory and multiple hepatocellular adenoma are associated with a higher BMI. Eur J Gastroenterol Hepatol 29:1183–1188 28704224 10.1097/MEG.0000000000000930

[19] Bunchorntavakul C, Bahirwani R, Drazek D, Soulen MC, Siegelman ES, Furth EE, Olthoff K, Shaked A, Reddy KR (2011) Clinical features and natural history of hepatocellular adenomas: the impact of obesity. Aliment Pharmacol Ther 34:664–674 21762186 10.1111/j.1365-2036.2011.04772.x

[20] Barte JC, ter Bogt NC, Bogers RP, Teixeira PJ, Blissmer B, Mori TA, Bemelmans WJ (2010) Maintenance of weight loss after lifestyle interventions for overweight and obesity, a systematic review. Obes Rev 11:899–906 20345430 10.1111/j.1467-789X.2010.00740.x

[21] Anastasiou CA, Karfopoulou E, Yannakoulia M (2015) Weight regaining: from statistics and behaviors to physiology and metabolism. Metabolism 64:1395–1407 26362728 10.1016/j.metabol.2015.08.006

[22] Arterburn DE, Telem DA, Kushner RF, Courcoulas AP (2020) Benefits and risks of bariatric surgery in adults: a review. JAMA 324:879–887 32870301 10.1001/jama.2020.12567

[23] Mocanu V, Mihajlovic I, Dang JT, Birch DW, Karmali S, Switzer NJ (2021) Evolving trends in North American gastric bypass delivery: a retrospective MBSAQIP analysis of technical factors and outcomes from 2015 to 2018. Obes Surg 31:151–158 32761442 10.1007/s11695-020-04889-3

[24] Goonawardena J, Ratnayake C, Cheung KT, Fox A (2020) Should bariatric surgery be offered for hepatocellular adenomas in obese patients? Surg Obes Relat Dis 16:2117–2124 32771427 10.1016/j.soard.2020.06.043

[25] Gevers TJG, Marcel Spanier BW, Veendrick PB, Vrolijk JM (2018) Regression of hepatocellular adenoma after bariatric surgery in severe obese patients. Liver Int 38:2134–2136 30025198 10.1111/liv.13934

[26] Khaoudy I, Rebibo L, Regimbeau JM (2018) Is bariatric surgery a potential new treatment for large inflammatory hepatocellular adenomas in obese patients? Surg Obes Relat Dis 14:535–538 29555032 10.1016/j.soard.2018.01.006

[27] Dokmak S, Belghiti J (2015) Will weight loss become a future treatment of hepatocellular adenoma in obese patients? Liver Int 35:2228–2232 26216699 10.1111/liv.12925

[28] von Elm E, Altman DG, Egger M, Pocock SJ, Gøtzsche PC, Vandenbroucke JP (2007) The strengthening the reporting of observational studies in epidemiology (STROBE) statement: guidelines for reporting observational studies. Ann Intern Med 147:573–577 17938396 10.7326/0003-4819-147-8-200710160-00010

[29] Munn Z, Barker TH, Moola S, Tufanaru C, Stern C, McArthur A, Stephenson M, Aromataris E (2020) Methodological quality of case series studies: an introduction to the JBI critical appraisal tool. JBI Evid Synth 18:2127–2133 33038125 10.11124/JBISRIR-D-19-00099

[30] Bonanomi G, Mandalà L, Maruzzelli L (2010) Laparoscopic staged adjustable gastric banding and liver resection in morbidly obese patient. Obes Surg 20:1186–1190 18830783 10.1007/s11695-008-9700-y

[31] Dantas ACB, Santo Filho MA, Jeismann VB, de Faria LL, Muniz RR, Rocha MS, Herman P, Santo MA (2021) Long-term complete remission of large hepatocellular adenoma after bariatric surgery. Obes Res Clin Pract 15:300–302 33766489 10.1016/j.orcp.2021.03.008

[32] Dauleh H, Soliman A, Haris B, Khalifa A, Al Khori N, Hussain K (2022) Case report: hepatic adenomatosis in a patient with Prader-Willi syndrome. Front Endocrinol (Lausanne) 13:826772 35355562 10.3389/fendo.2022.826772 PMC8959895

[33] Aziz H, Underwood PW, Gosse MD, Afyouni S, Kamel I, Pawlik TM (2024) Hepatic adenoma: evolution of a more individualized treatment approach. J Gastrointest Surg 28:975–982 38521190 10.1016/j.gassur.2024.03.010

[34] Frenette C, Mendiratta-Lala M, Salgia R, Wong RJ, Sauer BG, Pillai A (2024) ACG clinical guideline: focal liver lesions. Am J Gastroenterol 119:1235–1271 38958301 10.14309/ajg.0000000000002857

[35] Oudmaijer CAJ, Berk KA, van der Louw E, de Man R, van der Lelij AJ, Hoeijmakers JHJ (2022) KETOgenic diet therapy in patients with HEPatocellular adenoma: study protocol of a matched interventional cohort study. BMJ Open 12:e053559 35168973 10.1136/bmjopen-2021-053559 PMC8852750

[36] Anhê FF, Zlitni S, Zhang SY, Choi BS, Chen CY, Foley KP, Barra NG, Surette MG, Biertho L, Richard D, Tchernof A, Lam TKT, Marette A, Schertzer J (2023) Human gut microbiota after bariatric surgery alters intestinal morphology and glucose absorption in mice independently of obesity. Gut 72:460–471 36008102 10.1136/gutjnl-2022-328185 PMC9933168

[37] Coen PM, Tanner CJ, Helbling NL, Dubis GS, Hames KC, Xie H, Eid GM, Stefanovic-Racic M, Toledo FG, Jakicic JM, Houmard JA, Goodpaster BH (2015) Clinical trial demonstrates exercise following bariatric surgery improves insulin sensitivity. J Clin Invest 125:248–257 25437877 10.1172/JCI78016 PMC4382227

[38] Elhelw O, Ragavan S, Majeed W, Alkhaffaf B, Mohammed N, Senapati S, Ammori BJ, Robinson JA, Syed AA (2024) The impact of bariatric surgery on liver enzymes in people with obesity: a 5-year observational study. Surgeon 22:e26–e33 37567846 10.1016/j.surge.2023.07.006

[39] Nostedt JJ, Switzer NJ, Gill RS, Dang J, Birch DW, de Gara C, Bailey RJ, Karmali S (2016) The effect of bariatric surgery on the spectrum of fatty liver disease. Can J Gastroenterol Hepatol 2016:2059245 27777925 10.1155/2016/2059245 PMC5061986

[40] Sasaki A, Nitta H, Otsuka K, Umemura A, Baba S, Obuchi T, Wakabayashi G (2014) Bariatric surgery and non-alcoholic fatty liver disease: current and potential future treatments. Front Endocrinol (Lausanne) 5:164 25386164 10.3389/fendo.2014.00164 PMC4209858

[41] Drucker DJ (2018) Mechanisms of action and therapeutic application of glucagon-like peptide-1. Cell Metab 27:740–756 29617641 10.1016/j.cmet.2018.03.001

[42] Wang L, Berger NA, Kaelber DC, Xu R (2024) Association of GLP-1 receptor agonists and hepatocellular carcinoma incidence and hepatic decompensation in patients with type 2 diabetes. Gastroenterology 167:689–703 38692395 10.1053/j.gastro.2024.04.029 PMC12294230

[43] Wang XC, Gusdon AM, Liu H, Qu S (2014) Effects of glucagon-like peptide-1 receptor agonists on non-alcoholic fatty liver disease and inflammation. World J Gastroenterol 20:14821–14830 25356042 10.3748/wjg.v20.i40.14821 PMC4209545

[44] Gutierrez AD, Gao Z, Hamidi V, Zhu L, Saint Andre KB, Riggs K, Ruscheinsky M, Wang H, Yu Y, Miller C 3rd, Vasquez H, Taegtmeyer H, Kolonin MG (2022) Anti-diabetic effects of GLP1 analogs are mediated by thermogenic interleukin-6 signaling in adipocytes. Cell Rep Med 3:100813 36384099 10.1016/j.xcrm.2022.100813 PMC9729831

[45] Imbus JR, Voils CI, Funk LM (2018) Bariatric surgery barriers: a review using Andersen’s model of health services use. Surg Obes Relat Dis 14:404–412 29249585 10.1016/j.soard.2017.11.012 PMC6039385

[46] Iuzzolino E, Kim Y (2020) Barriers impacting an individuals decision to undergo bariatric surgery: a systematic review. Obes Res Clin Pract 14:310–320 32674935 10.1016/j.orcp.2020.07.001

[47] Martin AN, Marino M, Killerby M, Rosselli-Risal L, Isom KA, Robinson MK (2017) Impact of Spanish-language information sessions on Spanish-speaking patients seeking bariatric surgery. Surg Obes Relat Dis 13:1025–1031 28286039 10.1016/j.soard.2017.01.009

[48] Murtha JA, Alagoz E, Breuer CR, Finn A, Raffa SD, Voils CI, Funk LM (2022) Individual-level barriers to bariatric surgery from patient and provider perspectives: a qualitative study. Am J Surg 224:429–436 34963509 10.1016/j.amjsurg.2021.12.022 PMC9218004

[49] Westerveld D, Yang D (2016) Through thick and thin: identifying barriers to bariatric surgery, weight loss maintenance, and tailoring obesity treatment for the future. Surg Res Pract 2016:8616581 27314062 10.1155/2016/8616581 PMC4893581

