# Supplementary material for: Hepatic adenoma regression after bariatric surgery: a case series and systematic review
Source: Surg Endosc. 2025 Nov 10;40(2):1147–56. doi: 10.1007/s00464-025-12350-8 (PMC12881035; doi:10.1007/s00464-025-12350-8)
Supplement: Supplementary file 2 — Supplementary file2 (DOCX 28 KB) [file 464_2025_12350_MOESM2_ESM.docx]

Supplementary Table 2

Search terms used on screened patient records Keywords

Hepatic Adenoma Adenoma

Liver Lesion Liver Mass Hemangioma Liver cyst Liver tumor

Focal Nodular Hyperplasia

FNH

Hepatocellular

Hepatocellular adenoma Hepatocellular lesion

ICD Codes

# ICD-9 211.5/ICD-10 D13.4

Benign neoplasms of liver including HA.

# ICD-9 235.3/ICD-10 D37.6

Neoplasm of uncertain behavior of liver, GB, and bile duct.

# ICD-9 793.9/ICD-10 R93.2

Abdominal findings on diagnostic imagining of liver and biliary tract.

# ICD-9 211.5/ICD-10 D13.4

Benign neoplasms of liver including HCA.

# ICD-9 573.9/ICD-10 K76.9

Liver disease, unspecified.
