# Supplementary material for: Hepatic adenoma regression after bariatric surgery: a case series and systematic review
Source: Surg Endosc. 2025 Nov 10;40(2):1147–56. doi: 10.1007/s00464-025-12350-8 (PMC12881035; doi:10.1007/s00464-025-12350-8)
Supplement: Supplementary file 3 — Supplementary file3 (DOCX 21 KB) [file 464_2025_12350_MOESM3_ESM.docx]

**Supplementary Table 3**

**JBI Quality Assessment**

**Case Report**

| **Author** | **Q1** | **Q2** | **Q3** | **Q4** | **Q5** | **Q6** | **Q7** | **Q8** | **Total** | **%** | **Quality** |
| --- | --- | --- | --- | --- | --- | --- | --- | --- | --- | --- | --- |
| **Bonanomi** | Y | Y | Y | Y | Y | Y | N/A | Y | 7/7 | 100% | High |
| **Dantas** | Y | Y | Y | Y | Y | Y | N/A | Y | 7/7 | 100% | High |
| **Dauleh** | Y | N | Y | Y | Y | U | N/A | Y | 5.5/7 | 79% | High |
| **Khaoudy** | Y | Y | Y | Y | Y | Y | N/A | Y | 7/7 | 100% | High |

**Case Series**

| **Author** | **Q1** | **Q2** | **Q3** | **Q4** | **Q5** | **Q6** | **Q7** | **Q8** | **Q9** | **Q10** | **Total** | **%** | **Quality** |
| --- | --- | --- | --- | --- | --- | --- | --- | --- | --- | --- | --- | --- | --- |
| **Gevers** | Y | Y | Y | Y | Y | Y | Y | Y | Y | N/A | 9/9 | 100% | High |

Y: Yes (1)

N: No (0)

U: Unclear (0.5)

**Case Report**

1. Were patient’s demographic characteristics clearly described?
2. Was the patient’s history clearly described and presented as a timeline?
3. Was the current clinical condition of the patient on presentation clearly described?
4. Were diagnostic tests or assessment methods and the results clearly described?
5. Was the intervention(s) or treatment procedure(s) clearly described?
6. Was the post-intervention clinical condition clearly described?
7. Were adverse events (harms) or unanticipated events identified and described?
8. Does the case report provide takeaway lessons?

**Case Series**

1. Were there clear criteria for inclusion in the case series?
2. Was the condition measured in a standard, reliable way for all participants included in the case series?
3. Were valid methods used for identification of the condition for all participants included in the case series?
4. Did the case series have consecutive inclusion of participants?
5. Did the case series have complete inclusion of participants?
6. Was there clear reporting of the demographics of the participants in the study?
7. Was there clear reporting of clinical information of the participants?
8. Were the outcomes or follow up results of cases clearly reported?
9. Was there clear reporting of the presenting site(s)/clinic(s) demographic information?
10. Was statistical analysis appropriate?
